# Disability Community Perspectives on Participation in Research and Studying Positive Health

**DOI:** 10.3390/children13030430

**Published:** 2026-03-20

**Authors:** Melissa M. Murphy, Judy L. Aschner, Paige S. Ryals, Ana Joselyn Barahona, Jennifer Lyman, Ashley Harris Whaley, Rachel Byrne, Nathalie L. Maitre

**Affiliations:** 1 Division of Neonatology, Emory University School of Medicine, Atlanta, GA 30322, USA; melissa.murphy@cuanschutz.edu (M.M.M.); ana.joselyn.barahona@emory.edu (A.J.B.); 2 Center for Discovery and Innovation, Department of Pediatrics, Hackensack Meridian School of Medicine, Nutley, NJ 07110, USA; judy.aschner@hmhn.org; 3 Cerebral Palsy Foundation, New York, NY 10019, USA; jennifer.lyman@yourcpf.org (J.L.); ashley.harris.whaley@yourcpf.org (A.H.W.); rachel.byrne@yourcpf.org (R.B.); 4 Children’s Healthcare of Atlanta, Atlanta, GA 30329, USA

**Keywords:** Environmental influences on Child Health Outcomes (ECHO), childhood disability, positive health, research participation, disability community, disability perspectives

## Abstract

**Highlights:**

**What are the main findings?**

- Despite >80% of people with lived experience of disability endorsing health outcomes research, only about a quarter (1/4) of people report participating in non-disability-specific research and about a quarter (1/4) to a third (1/3) report having been excluded from research because of a disability.
- Conceptualizations of positive health outcomes that include “absence of disease” need to be reframed in a way that accounts for childhood-onset disability—i.e., biological impairments/conditions that alter one’s power and capacity to act in the world.

**What are the implications of the main findings?**

- Reframing positive health as a research outcome in the context of childhood-onset disability may enhance its relevance to the disability community.
- Researchers can improve disability community inclusion in research through specific actions to strengthen relevance, generalizability, and impact of research.

**Abstract:**

Background/Objectives: Disability affects ~15.7 million children and ~67 million adults in the US, yet these individuals are typically under-represented in clinical research. Clinical research has increasingly broadened its focus on health outcomes to include “positive health,” which reflects the capacity of an individual to adapt to challenges and the absence of disease. Methods: A mixed-methods approach is used to investigate disability community perspectives on research inclusion and the use of positive health as an outcome in the context of childhood-onset disability. Results: Nationally, about one-fourth (1/4) of adults with disabilities and parents/caregivers reported participating in *non-disability-specific* research; overall, ~23% of adults and ~30% of parents/caregivers report exclusion because of disability, despite >80% endorsing health outcomes research. Disability stakeholders unanimously express the need to reframe positive health in a disability context, provide guidance on how to reframe it as a research outcome, and offer a roadmap for improving research inclusion. Conclusions: A paradigm shift in how positive health is framed may enhance its relevance to disabilities. An action plan for researchers is derived as a pragmatic approach to strengthen the relevance, generalizability and impact of their research.

## 1. Introduction

Over the past decade, clinical research has transformed from examining health outcomes exclusively related to illness and disease to a broader focus on “positive health” [1], which encompasses constructs like wellbeing, resilience, and the capacity to adapt to challenges that lead to health assets or the absence of disease [2,3]. In 2016, the National Institutes of Health Environmental influences on Child Health Outcomes (ECHO) Program, studying the developmental origins of child health, adopted positive health as one of its five health outcomes [1]. At a population level, a focus both on the prevention of illness and optimizing health has the potential to improve and accelerate overall health and wellness. However, the conceptualizations of positive health that include “absence of disease” present an inherent conflict when applied in the context of childhood onset and neurodevelopmental disabilities. Generalizable research then requires understanding how positive health is perceived by people with lived experience of childhood-onset disability, along with barriers to research participation in this context. Thus, the present study seeks to fill this knowledge gap and derive actionable recommendations for researchers interested in positive health and disability that is informed by disability stakeholders.

Definitions of disability vary, with most agreeing on the presence of a long-term impairment that interacts with a person’s environment and hinders their participation in society on an equal basis as others [4,5,6]. Childhood-onset disabilities are a subset of disabilities that originate prior to age 18 and are associated with physical, mental, or developmental impairments that last through adulthood [7,8,9]. Estimates of disability prevalence vary in the US [10], with recent data suggesting that it affects 15.7 million children and 67 million adults [10,11]. Despite this prevalence, individuals with disabilities are typically under-represented in clinical research [12]. This under-representation is problematic because disability is most often handled by researchers as an “outcome to prevent or treat” rather than a condition in which health should be studied to be optimized. The present study focuses on systematically understanding the perspectives of caregivers of a child with a disability and adults with a childhood-onset disability as it relates to inclusion in research and “positive health” as a research outcome.

The conceptualizations of positive health vary with no single agreed-upon conceptual framework [3,13]. In part, a lack of agreement may reflect variability in the meaning of “health” and a general recognition that “health” as a construct may vary depending upon contextual factors, personal circumstances, and individual characteristics (as reviewed by [13]). One-dimensional conceptualizations of health (e.g., [14,15,16]) represent health along a single continuum from the presence to the absence of disease [3,14]. From this perspective, a current state of health is determined by myriad interactions of protective and risk factors [3]. As a result, positive health (e.g., wellbeing, resilience) represents the end of the continuum “far beyond the mere absence of disease” [3]. An alternative conceptualization recognizes that health and disease exist along two distinct continua. This two-dimensional framework allows for health to represent the additive effects of both health- and disease-related processes, defining positive health as “capacity” or “resources” to adapt to social, physical, and emotional challenges [3].

ECHO draws on this two-dimensional conceptualization by defining health as set of resources deployed to “adapt to environmental challenges, satisfy needs, and reach a person’s goals” (p. 298) alongside disease and illness [1]. It further situates this conceptualization within a systems-framework that recognizes the complex interactions of health and disease within a child’s physical, social, and family environment over time [1]. Thus, two complementary components are used to measure positive health in ECHO: subjective wellbeing and the positive child health index (PCHI) [17].

The biological impairments (“disease”) that are associated with childhood-onset disability mean that development for that child and his/her family inherently reflects the interaction of a biological impairment/condition (“disease”) and capacity to act and adapt in the world (“health”) with the physical and social environment around them. This notion of person–environment–disability interaction is exemplified in the International Classification of Function (ICF) framework, which is used as the main framework for conceptualizing interactions between health and disability for individuals with disabilities [18]. As such, understanding positive health in the context of childhood-onset disabilities provides an important opportunity to further develop conceptualizations of health. Yet research is lacking on how stakeholders in the disability community nationally and locally (e.g., disabilities advocates, families) perceive positive health as a health outcome.

The present study addresses this knowledge gap by investigating disability community perspectives on the use of positive health as an outcome in the context of childhood-onset disability. We also explore perceived barriers and facilitators of research inclusion that can be addressed to strengthen disability research programs. We then derive a pragmatic stakeholder-driven action plan for researchers, especially those who plan to use positive health as a research outcome.

## 2. Materials and Methods

### 2.1. Design

Grounded in a phenomenological approach, an explanatory sequential mixed-methods study design was applied: a quantitative national survey (Part I) was followed by the qualitative analysis of transcripts from disability advisory board discussions (Part II) to inform and extend survey results.

### 2.2. Ethical Review

Ethical review and approval were waived for this study due to the project being deemed as “not human subjects research” by the Hackensack Meridian Health IRB.

Part I—Non-identifiable national survey responses were collected to help inform research questions and gaps.

Part II—Qualitative analysis consisted of the secondary data analysis of transcripts from disability advisory board discussions, which followed a focus group-type format. The advisory board charge was to review ECHO materials, forms, and scripts and provide direct feedback to ECHO researchers related to disability community engagement. Feedback from the advisory board was compiled on a biannual basis to inform the project. Advisory board members were compensated for their time and effort on the advisory board. No compensation was provided for inclusion in secondary data analysis arising from the discussions, such as those presented here. Transcripts were de-identified (names replaced with a numeric code and any additional identifying information removed) by the advisory board facilitators prior to analysis by the research team.

### 2.3. Part I: National Survey

An investigator-designed survey about research participation was open from October to November 2022. The Cerebral Palsy Foundation (CPF) distributed the survey through channels reaching both the broader disability community and those with cerebral palsy. This effort included promotion through CPF social media platforms, website, and organizational email lists. To broaden participation beyond CP-specific audiences, we also asked partner organizations (e.g., Disability Reframed, American Association of People with Disabilities) and networks serving a range of disability communities to disseminate the survey through their newsletters, mailing lists, and social media channels. To access the survey, respondents needed to be: (1) an individual >18 years with a condition causing disability or (2) a parent/caregiver of a child <18 years with a condition causing disability (Table 1). To avoid duplication, the survey could be completed only once per IP address.

The survey consisted of 13 items and took ~10 min to complete (Supplementary File S1). Results from two survey questions about research inclusion are reported here: (1) Have you ever participated in a research study that was NOT meant to study your/your child’s disability? (2) Have you/your child ever been EXCLUDED from a research study because of a disability (physical, mental, emotional…)? Three additional questions, which are not reported in this paper, asked respondents to rate on a scale of 1 (low)–100 (high) the importance of including people with disabilities in research on environment impacts. Demographic information was also collected. A total of 350 adults and 86 caregivers answered the two target questions on the survey and provided demographic information.

### 2.4. Part II: Advisory Board Transcript Analysis

#### 2.4.1. Underlying Methodological Framework

Based on findings from the national survey about under-representation in research, part II applied Participatory Action Research (PAR) methodology [19,20]. In brief, PAR is a systematic, multi-step process involving: (1) selecting partner groups and a forum (setting the stage), (2) encouraging contributions and diverse perspectives during the forum (implementing), (3) summarizing collected responses looking for patterns, insights, and action steps (analyzing), and (4) seeking feedback from forum participants on patterns identified and future actions (circling back).

#### 2.4.2. Eligibility and Recruitment for ECHO Disability Advisory Board

The primary purpose of the advisory board was to advise investigators of an ECHO pregnancy cohort that are recruiting participants in New Jersey (NJ) and Illinois (IL) [21]. Therefore, convenience sampling was used to identify eligible participants in Illinois or New Jersey who were either (1) a parent/caregiver of a child 2–17 years, identified as having developmental disabilities (cerebral palsy, autism, intellectual/developmental, gastrointestinal, and/or vision/hearing impairment, with communication, feeding, or sleep-related issues), or (2) an adult with similar disability profiles.

The CPF reached out via phone and email to members of a wide social network of adults with cerebral palsy, families, therapists, and physicians. One social media post was used to generate more potential participants. Interested individuals were referred to the CPF team (AHW, JL), who reached out via email to establish initial contact.

Thirteen individuals met participation criteria for the advisory board. A matrix stratifying age, race/ethnicity and disability identified 12 individuals for the initial group. These individuals received an invitation and call for informed consent to participate in the advisory board. Of those approached, 100% participated, with ten selected and others wait-listed. One person stopped participating after the first meeting due to scheduling conflicts and was replaced by a person from the wait-list. Another person was unable to attend the meetings described here due to illness. In addition to these 9 members, two employees of the CPF with lived experience of disability were included in advisory board discussions by virtue of their involvement in the board meetings. Unlike the other advisory board members, they did not receive any compensation for participation. The result was 11 advisory board participants in the discussions described here.

#### 2.4.3. Participants

Advisory board participants who participated in discussions analyzed here were caregivers of a child with a disability (*n* = 7) and adults with disability lived experience (*n* = 4) (Table 1).

#### 2.4.4. Setting and Data Collection

Transcripts were obtained from multiple 90 min advisory board meetings held and recorded via Zoom. Content and structure were identical, consisting of an overview of the ECHO program and the goals of the ECHO pregnancy cohort in NJ and IL (20 min), the group’s charge for the session (5 min), open discussion of two questions (~30 min/question; Table 2), wrap up and homework (5 min). Results from the national survey were also used to inform discussion prompts for the ECHO disability advisory board.

An experienced facilitator (MMM) guided discussion with Principal Investigator (PI, NLM) monitoring the chat, allowing for the real-time member checking of synthesized data to increase data validity [22,23,24] and ensure data saturation. The two CPF participants were acquainted with the PI (NLM) and facilitator (MMM) prior to the initial meeting.

During the session, participants were encouraged to share their perspective either verbally or through the chat. The facilitator took fieldnotes and recorded impressions following the discussions. Afterwards, participants were invited to share additional thoughts by email or phone call.

### 2.5. Analysis

Quantitative: Descriptive statistics from the national survey used SPSS version 29 [25].

Qualitative: Cloud recordings of advisory board discussion were transcribed by Rev.com and chats exported as text. De-identified source data were uploaded into MAXQDA (VERBI Software, 2024) and combined for analysis. Following thematic analysis steps outlined by Braun and Clark [26], the analysis team (MMM, PSR, AJB) familiarized themselves with the source data. Using an inductive reasoning approach, they independently identified initial codes associated with each question. The analysis team met to discuss possible preliminary themes and re-reviewed source data to reach initial group consensus on preliminary themes. Source data, codes, and themes were then reviewed and discussed with project lead (NLM) to ensure themes were refined to best reflect group consensus on theme salience, interconnectivity of ideas expressed, and maximize robustness and validity of revised themes.

The all-female analysis team represents culturally and professionally distinct perspectives and works with the disability community in research and clinical contexts. MMM is an educational psychologist. PSR is a clinical research coordinator. AJB is a post-baccalaureate trainee preparing to be a clinician-scientist. NLM is a neonatologist, pediatrician and scientist with personal and professional expertise in CP.

Once the analysis team articulated and refined preliminary themes and associated action recommendations, they circled back to the advisory board for feedback during a subsequent board meeting. At the meeting, the analysis team lead (MMM) and PI (NLM) shared the themes and drafts of the figures presented in the discussion. The advisory board provided feedback on the themes and context to ensure themes and interpretation were representative of their perspectives. The result of the discussion was acknowledgement of how well the themes resonated with their lived experience and universal approval of the themes and action recommendations by advisory board participants. Consolidated Criteria for Reporting Qualitative Research (COREQ) best practice guidelines [27] are followed for analysis and reporting of results.

## 3. Results

### 3.1. National Survey

Overall, 26.0% (91/350) adults and 23.2% (20/86) parents/caregivers reported having participated in a study that was *not disability-specific* (i.e., a research study that was NOT meant to study your/your child’s disability?). A total of 22.6% (79/350) adults and 30.2% (26/86) caregivers reported being excluded from a study *because of* disability. Across the cohort, including people/children with disabilities in research studying the role of the environment on domains of health, quality of life and happiness was very important: median scores were 85/100, 90/100 and 82/100, respectively (IQRs were 60–100, 61–100, 59–100, respectively).

### 3.2. Advisory Board Transcript Analysis

Identified themes reflect consensus regarding the relevance and salience of the perspectives raised. Themes and subthemes, definitions, and relevant quotes illustrating the essence of each theme using the participants’ own words are summarized in Table 3.

#### 3.2.1. Perspectives on Positive Health

Two interconnected themes were identified. The first, *childhood disability is lifelong*, reflects the idea that conceptualizing positive health—specifically in terms of “absence of disease”—must be radically reconsidered within a childhood disability context. The second theme follows from the first: *disability reframes health outcomes*. Thus, disability lived experience interacts with wellbeing, resilience, and capacity reserve (to adapt to challenges) in ways that create unique experiences of these concepts in the disability community for both the individual and caregiver/family.

#### 3.2.2. Theme 1: Childhood Disability Is Lifelong

Subtheme: Altered exposure to developmentally “typical” experiences profoundly affects components of positive health.

Childhood disability often requires choices about participation in life events above and beyond those of the general population; a child’s chances to fully participate in formative activities are therefore challenged or limited by the effort involved or absence of adaptation. From the ICF framework perspective, “participation in life situations” is a core component of functioning, and by extension, it affects attaining positive health. To the extent that positive health arises from participation in life situations and environmental factors that facilitate or prevent participation, positive health is affected across the life course by disability in ways that are different from the general population.

Subtheme: Positive social connection moderates the impact of disability on positive health.

Affirming social connection promotes positive health both for people with disabilities and their caregivers by alleviating isolation. Building a network of positive connections to other people who share similar experiences, are accepting, and supportive is integrally related to wellbeing and resilience. For youth, school was identified as an important context for connection and support. However, not all social connections were described as protective. There was collective acknowledgement of the negative impact that lack of acceptance, support, and understanding in interactions with local communities was a threat to wellbeing and resilience, increasing feelings of isolation.

#### 3.2.3. Theme 2: Disability Reframes Positive Health Concepts

Although presented separately in the definition of positive health, wellbeing, resilience, and capacity reserve are inherently interconnected from a disability perspective:

*“How can individuals and families affected by disability aspire to the same wellbeing as others because the perspectives on what they think what wellbeing should be are skewed by other imperatives, judgement of people who have not experienced disability, and sometimes prejudice or pity.”*

(P6)

Subtheme: Wellbeing of the family system, not just the child, affects positive health.

In a disability context, caregiver and child wellbeing were inextricably linked and reciprocal, supporting the idea that positive health must consider the wellbeing of the family system. From an ICF perspective, participants reflected that family systems and member wellbeing are environmental factors that can facilitate or compromise positive health for people with disabilities. When wellbeing is at risk, “worry and fear” can result. Along with mental health-related wellbeing, two other aspects of wellbeing integral to positive health were raised in the discussion. The word “solidarity” was used to refer to the shared experiences of disability associated with wellbeing and “a sense of… emotional satisfaction” from “knowing that there’s other people that have the same common experiences as you” (P3). This sentiment was consistent with social connection (Theme 1).

A second related aspect of wellbeing was the awareness that economic factors can determine “opportunity… to get involved in some programs… to experience that solidarity…” (P3). Financial costs of medical care, equipment, and accessibility were described as compounded by social risk factors, such as food, housing, or income insecurity—financial hardship worsening unmet social needs. Even when social risks are mitigated, disability care needed to achieve positive health was still described as associated with financial and emotional costs.

Subtheme: Resilience is a concept compelled by the world around disabled people complicating its relationship to positive health outcomes.

In the literature, resilience is typically characterized as capacity to adapt and grow following an traumatic event [28]. As such, it is an active process distinguished from recovery by the rapid return to pre-event levels of distress or better [29,30]. However, every participant attached negative connotations to the concept of resilience. They focused on resilience as a state of being that is “forced on disabled people” (P9) by the world around them. The same lack of a choice to be resilient was expressed in relation to children/adults with impairments as well.

This consistently negative perception is in stark contrast to the positive connotation the surrounding culture places on resilience. This dissonance was described as the need to “balance” between expectations of resilience required by the outside world and a lack of choice over whether to be or to stay impaired.

Subtheme: Disability lowers the baseline capacity reserve for children/adults and caregivers.

Baseline level of capacity is altered by the presence of chronic disability, which in turn impacts definitions of positive health. Specifically, there was general agreement that the nature of disability involves a myriad of complex tasks and coordination that require energy investments to start, sustain, and navigate. One caregiver described the experience as “micro wounds” which over time lead to “death by a thousand cuts” (P1).

For some, this alteration in baseline capacity resulted in what they referred to as constantly being in “survival mode,” which makes it “really hard to even think about positive health, wellbeing, resilience” (P8). When in survival mode, there is limited to no reserve of capacity for function, interactions or decision-making.

The baseline capacity reserve was specific to each person depending on factors including disability severity, social support availability, and financial strain. However, there was agreement that capacity reserve affects wellbeing and attainment of positive health.

### 3.3. Perspectives on Barriers and Facilitators to Participation and Obtaining Information About Positive Health Outcomes

Barriers and facilitators are summarized in Table 4. Of note, facilitators often included improvements that led to overcoming barriers [31]. Because of this dual role, the results that follow focus on facilitators.

Responses aligned with four facilitators to increase the feasibility of disability community engagement in research. Community education focuses broadly on the world around the child/family. Remaining facilitators focus on family-centric needs for connection, financial compensation, and resources.

*Community education*, providing the community (in this case researchers and research entities, but also healthcare and social systems) with a “baseline education around disability,” (P9) was recognized as a critical component of positive health, overcoming ableism, and facilitating study recruitment and information gathering.

*Opportunity for connection* with others who are accepting was identified as a key aspect of positive health and facilitator of information gathering in the disability community, especially because of its potential to overcome perceived isolation (e.g., described as a feeling that “nobody understands”). Participants expressed wanting “advocates” and professionals who understand disability perspectives.

*Resource needs* were identified to reduce burden of and increase capacity to participate in community-centric activities, including research. Addressing these needs may alleviate capacity limitations, unprocessed trauma, and internalized ableism that impede reporting and evaluating positive health. Participants expressed the need for:

(1) *Practical/logistical* resources (e.g., help navigating the healthcare system), emphasizing the value of involvement with and resources from caring professionals with broad knowledge of opportunities (e.g., “… what you can do is ask these families…, can we help you in some type of way? … [for example I] got in tune with this program through my child’s therapist that we don’t even see no more. [P10]”).
(2) *Information/education* (e.g., about disability and how any study outcomes relate to disability), including recognition of training/education needs for parents (e.g., “on how to raise a child with disabilities…”) and children (e.g., “make a class about disabilities in school systems” [P5]).
(3) *Psychological supports* (e.g., mental health and coping resources) expressed by a caregiver as “You could offer families, [the opportunity of] just expressing what they really feel… A lot of… families hold what they’re feeling in…” (P10). In the absence of support, unprocessed trauma associated with disparity may represent a participation barrier.
(4) *Financial compensation* to help defray added time and energy burden associated with participation, as one caregiver stated, “money is important” (P8). Throughout the discussion, financial compensation was related to the constraints that disability places on capacity to achieve participation goals and associated positive health.

## 4. Discussion

**Figure 1:**
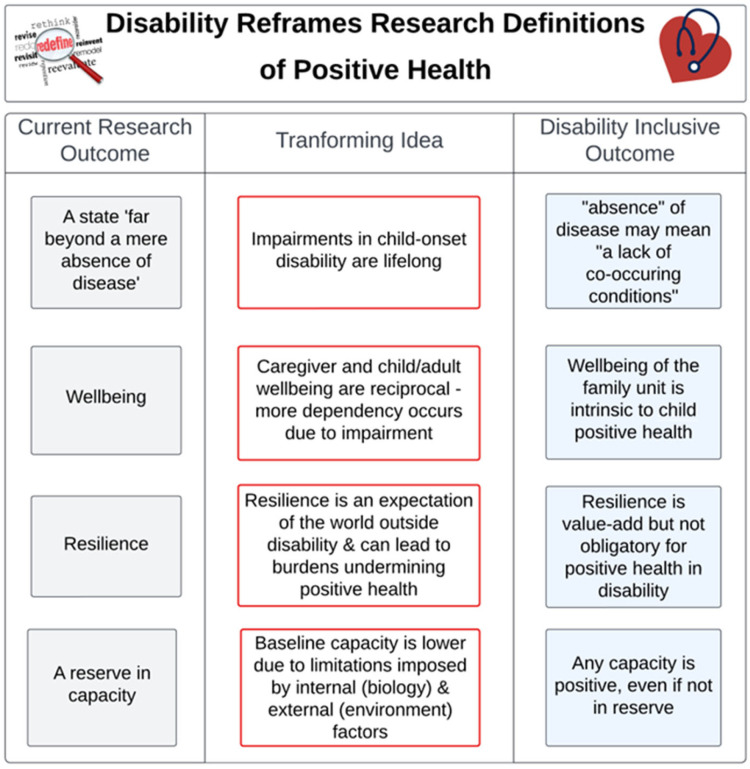
This figure illustrates the four transforming ideas to drive strategic change extracted from the mixed method analysis. Reframing the concept of positive health to be more inclusive of children/people with disabilities and their caregivers may compel learning within researcher and research participant communities. This learning can catalyze development of connection agents and platforms, ultimately applying concrete tactics to driving new initiatives that empower communities to more effectively participate in research.

The present study used a mixed-methods design to engage the disability community in research designed to encourage dialog related to positive health and research participation. Findings from our national survey demonstrate that reported inclusion of people with disabilities in non-disabilities-specific research is low, and people with disabilities often report being excluded from such research. Conversely, they think being included in research that examines interactions between environment and health, quality of life and happiness is important. We explored these results in detail through discussions with stakeholders serving as part of a disability advisory board for an ECHO pregnancy cohort. These stakeholders provided guidance on how to interpret the research outcome of “positive health” in a way that empowers people with disability lived experience (Figure 1) and provide a roadmap for what to do/avoid for improved inclusion in research assessing and promoting positive health. 

### 4.1. Reframing Positive Health

The unanimous consensus from the disability stakeholder group was a need to ensure that the conceptualization of positive health accounts for disability—that is, the biological impairments/conditions that alter one’s power and capacity to act in the world. This perspective supports the context validity ECHO’s conceptualization of positive health emphasizing the environment interacting with health along two dimensions: capacity and resources to adapt (health) and biological impairments resulting in disease [1]. Themes evident in the discussion related to absence of disease, wellbeing, resilience and capacity helped to contextualize the complex interactions among the environment, health, and biological impairment in disability.

#### 4.1.1. Absence of Disease

Disability stakeholders noted that the lifelong “impairments that alter function” inherent to childhood-onset disability interact both with efforts to promote health and the physical, social, and family environment. Limitations, therefore, arise from intrinsic (biological impairments in body or brain) and extrinsic (barriers presented by external factors). These impairments limited the autonomy of individuals with disability to act in the world as well the capacity of individuals and caregivers to adapt to the world around them, which was associated with a strong sense of injustice. As described by stakeholders, this experience aligns with two-dimensional conceptualizations of positive health [1,3] and highlights the importance of exploring health outcomes over time in the context of person-environment interactions [1].

#### 4.1.2. Resilience

Two-dimensional conceptualizations of positive health, including the Health Assets Model, incorporate the ability to resist adversity (i.e., resilience) as an important factor in positive health [3,32,33,34]. Disability stakeholders in the present study acknowledged the need for resilience while at the same time expressing it as “a concept compelled by the world around disabled people.” This interpretation of resilience through a lens of perceived injustice may account for the negative emotional valence present in participant responses. As defined, the ability to recover and grow from adversity that characterizes resilience relies on autonomy, the ability to make your own decisions rather than being told what to do. However, participants in the present study strongly expressed the ways in which their (or their child’s) impairment and the world around them deny that freedom of choice to do anything other than continuously recover and grow from adversity. As such, the conceptualizations of positive health that equate health with resilience may fail to recognize the unrelieved burden on people with disabilities and their caregivers to fit into a framework defined by freedom of choice they do not actually have. This notion is exemplified in the present study by stakeholder sentiment that they cannot overcome a condition that exists without them choosing it, as it is inherent because of an attribute of their own—or their child’s—body or brain.

The idea that “resilience is expected” expressed in the current study is consistent with findings from other under-represented groups [35]. Rather than easing unrelenting adversity, pressure to be more resilient can lead to burden and undermine efforts to support positive health [35]. This characterization of the dual nature of resilience captures the emotional essence of the discussion in the present study. From a research perspective, the implication of this observation is the need to rethink the frameworks used to understand and study resilience among the disability community.

#### 4.1.3. Wellbeing and Reserve in Capacity

The consensus among disability stakeholders in the present study was that “disability lowers the baseline capacity reserve for child/adult and caregiver.” Capacity reserve here referred to the ability/resources to adapt to social, physical, and emotional challenges [3]. Supporting this perspective were descriptions such as “everything is a fight,” “micro-wounds”, and “survival mode” that amplify the experience that “dis-ability” does not equate to “ability” in terms of opportunities and rights. As such, the idea of a “reserve in capacities” was perceived negatively due to the extent to which capacity is taxed by the surrounding world. Stakeholder descriptions of the complex, chronic, and sustained interaction of disability, environmental factors, and health outcomes are consistent with conceptualizations of positive health as multi- rather than uni-dimensional [1,3,18]. They further suggest the importance of considering the ways in which the experience of disability may mediate or moderate perceived wellbeing and health outcomes.

Also, evident in stakeholder discussions was the extent to which the effects of depleted capacity pervade the other aspects of positive health, thereby challenging caregiver and child/adult wellbeing and resilience. Indeed resilience, defined as the capacity of a person to adapt and grow despite adversity, sustain competence under stress, and recover from trauma [28], implies that the degree to which capacity is taxed is a key factor when evaluating positive health overall, and wellbeing and resilience specifically.

### 4.2. Roadmap: Reframe, Learn, Connect, Empower

**Figure 2:**
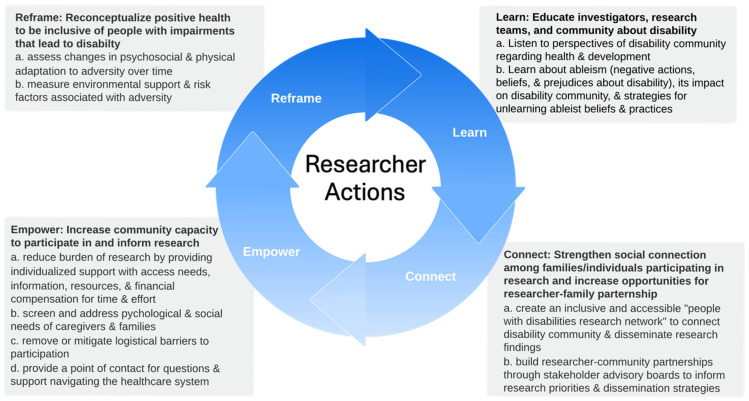
Roadmap for researcher actions.

Acknowledging national survey results suggesting limited participation in both general research and disability-specific research, disability stakeholders in the present study identified four actions to improve representativeness of disability in studies to assess and promote health in the community (Figure 2). These actions align with core ethical principles of human-centric research: respect for persons exemplified through autonomy and capacity, beneficence, and justice [36]. They are also consistent with stakeholder perspectives reported in prior studies of barriers and facilitators to research participation in cerebral palsy [20,37]. Acknowledging that the stakeholder advisory board represented both CP and non-CP disability, the convergence of findings suggests more similarities than differences in the childhood-onset disability community with regard to the impact of disability on health.

#### 4.2.1. Reframe: Reconceptualize Positive Health to Be Inclusive of People with Impairments That Lead to Disability

This first suggested action is evident in stakeholder discussions of health described previously (Figure 1). Ensuring the relevance and significance of health in the context of disability is needed to ensure the fair distribution of research burdens and benefits consistent with the principle of justice.

#### 4.2.2. Learn: Educate Investigators, Research Teams, and Community About Disability

Participants expressed the importance of researchers both listening to the perspectives of the disability community about health and educating themselves about ableism. Community-based participatory research methods, such as focus groups, are a formal way to engage the disability community. Less formal ways include conversing with study participants about their research experiences and areas of health that matter to them. Maximizing benefits to participants by understanding what is important to them and diminishing harms by minimizing ableist beliefs and practices exemplify the practice of beneficence.

#### 4.2.3. Connect: Strengthen Social Connection Among Families/Individuals Participating in Research and Increase Researcher–Family Partnerships

Consistent with other findings [20] and studies of social connection [38,39,40], participants emphasized the importance of social connection. Establishing local and regional disabilities research networks is one way to connect the disability community to each other. It may also provide pathways to enhance community-based recruitment, disseminate research findings, and inform researcher–community partnerships, such as advisory boards and patient-partners. Enhancing connections in these ways epitomizes the principle of beneficence by increasing and distributing benefits of knowledge gained through research to persons within the disability community and ensuring research addresses problems that matter to participants in ways that are meaningful.

#### 4.2.4. Empower: Increase Community Capacity to Participate in and Inform Research

Like connection, the importance of building trust between researchers and disability community is consistent with published findings [20]. Environmental and biologically imposed limitations associated with disability lower baseline capacity to participate in research. Principles of justice and respect for persons prompt consideration of ways to reduce participation burden (e.g., by removing barriers and amplifying facilitators) that make participation a true choice rather than an automatic exclusion. Suggestions offered by participants include screening and addressing psychological and social needs and providing a point of contact for navigating the healthcare system. Moreover, research programs that emphasize co-creation of research priorities and outcomes in partnership with community members can magnify research impacts and empower community members.

### 4.3. Limitations and Strengths

Studies that utilize community-based research approaches can help empower the community and lead to positive change yet necessitate smaller sample sizes. However, they also allow for more thorough, nuanced, and in-depth exploration of potentially sensitive topics than surveys, which are not without their own limitations (e.g., perception of being non-personal and difficulty interpreting the meaning behind responses). By necessity, this community-based qualitative approach limited the number of participants. Additional community-based advisory groups for research will strengthen the generalizability of these findings and ensure thematic saturation on the topic of health in disability.

Convenience sampling approaches, like the ones used in the present study, do not reflect the perspective of persons who are unable to participate because of accessibility-related concerns, practical limitations (e.g., time, lack of awareness of opportunity), and/or other research barriers. The national survey was conducted online with accessibility accommodations (e.g., screen reader compatibility, alt text for images) and targeted a broad range of disabilities, including but not limited to cerebral palsy. Together platforms used for survey distribution had the potential to reach >150,000 individuals with a disability. However, the broad sampling approach did not allow calculation of a response rate to know what percentage of the population received the survey and responded. The survey also did not ask about the nature of the disability making it impossible to distinguish etiology, type, or severity of disability. Ongoing efforts are needed to document perspectives of people with disabilities using validated survey instruments across a broader range of geographical regions, experience levels, ages, and phenotypic characteristics. Differences in phenotypic characteristics of disability may be important mediators or moderators of environmental effects on health outcomes.

Advisory board composition was intended to be the representative of childhood-onset disability rather than specific to a specific diagnosis. Efforts were made to ensure representation: of members, 4/6 had lived experience of cerebral palsy. CP as a physical disability has not previously been the focus of ECHO cohorts as has been the case with intellectual and developmental disability (IDD) and autism spectrum disorders (ASDs). In addition to CP, IDD, ASD, sensory impairment, and complex medical care were represented across the advisory board. Despite this varied representation, caution is needed in generalizing the findings in the broader context of all disability, and additional research using community-based approaches can ensure validity and reliability of results across different types of disability.

Another consideration when interpreting these results is that advisory board membership was determined by geographical regions with ECHO pregnancy cohort representation. State-level differences in disability policy and resources may limit transferability of findings to the broader disability community and should be considered in any disability-specific policy work or research. Advisory board discussions did acknowledge state-level concerns; however, the themes identified here were universally agreed upon by advisory board members in discussion and upon review.

The design of the disability advisory board, distinct from this study and through which secondary data were obtained, involved Zoom-based meetings to reduce burden and increase capacity to participate by providing compensation for time, virtual meetings, facilitating assistive communication usage, and meetings timed to fit availability. Of note, two participants in advisory board discussions were employees of CPF and acquainted with the PI and meeting facilitator. These individuals participated in advisory board discussions by virtue of their involvement in the board meetings, and so were not excluded from the discussion transcripts, nor did they receive any compensation for participation in advisory board discussions. Neither individual reported to the PI or meeting facilitator and transcripts were de-identified, which reduced the possibility of associating responses with specific board members.

## 5. Conclusions

Findings in the present study suggest that the multi-dimensional conceptualizations of health align with disability stakeholder perceptions of what it means to have “positive health” and support the context validity of frameworks for positive health like the one used in ECHO. At the same time, to enhance the relevance of research on positive health for people with disabilities, a paradigm shift is needed to ensure that health is framed in the context of disability and its interaction with the environment to produce health outcomes. Disability stakeholders in the present study provide the reason for and actions to accomplish this paradigm shift that align with ethical principles of human research. Researchers can consider these actions as a pragmatic approach to strengthen the relevance, generalizability and impact of their research. Moreover, engaging disability stakeholders in all aspects of research from question formulation through interpretation of results is critical to ensure the accuracy of interpretations in a disability context and engage the disability community as partners in research and policy decisions arising from that research.

## Figures and Tables

**Table 1:** Characteristics of survey participants: number of total respondents reporting.

	Adults	Caregivers
Survey	*n* = 350	*n* = 86
Gender (*n* females, not reported)	171, 15	43, 9
Age range of person with disability		
18–29 years	46	9
30–44 years	120	22
45–60 years	127	40
>60 years	42	6
Not reported	15	9
Income		
$0–$9999	83	18
$10,000–$24,999	81	17
$25,000–$49,999	73	21
$50,000–$74,999	48	7
$75,000–$99,999	15	5
$100,000+	18	4
Prefer not to answer	32	14
Region		
East North Central	53	12
East South Central	29	10
Middle Atlantic	48	9
Mountain	22	1
New England	21	2
Pacific	26	5
South Atlantic	71	20
West North Central	24	8
West South Central	40	8
Not reported	16	11
Disability Advisory Board	*n* = 4	*n* = 7
Gender (*n* females)	2	6
Relation to person (*n* mothers)		6
Race (*n*, Black)	1	3
Ethnicity (*n*, Hispanic)	1	1
Age range of person with disability (years)	22–32	3–20
Diagnosis (*n*, CP)	4	6
Employed	2	5

**Table 2:** Discussion Questions.

1. How is the concept of positive health stated below relevant/meaningful in the context of disability in childhood? For example: Do these terms resonate with you? What would you add (experiences, feelings, or events) under these categories to make them more relevant? Positive health can be defined in 4 ways: A state ‘far beyond a mere absence of disease’ Wellbeing Resilience A reserve in capacities
2. As we include more children with disabilities in ECHO from the earliest age and follow them into adulthood, what are things we could do or ask to make obtaining information on positive health outcomes more feasible? What do you see as obstacles or barriers in reporting or evaluating positive health in these families?

**Table 3:** Themes and subthemes arising from disability stakeholder perspectives on the meaningfulness of positive health defined as “a state ‘far beyond a mere absence of disease’, wellbeing, resilience, and a reserve in capacities”.

Theme/Subtheme	Definition	Examples
Childhood disability is lifelong	Refers to chronic nature of disability and/or notion that the absence of disease is unattainable	“[my child is] always going to have cerebral palsy and that’s going to make her feel fatigued and it’s going to impact her in ways that doesn’t impact our other kids” (P1).
Altered exposure to developmentally “typical” experiences profoundly affects components of positive health	Refers to the lack or limiting of enriching experiences due to logistical, physical, and emotional considerations related to disability	“[At a younger age, my child] really couldn’t do… things that other children were able to do. And still to this day, she cannot do… things that other children can do, like cheerleading. She wants to be a cheerleader, but unfortunately she can’t. Her muscles were tightened and stiff… (P11). “Why can’t [they] be accepted as a child… all kids are different, but they’re still kids and they should never have to feel like they’re not a part of society” (P11).
Positive social connection moderates the impact of disability on positive health	Refers to social connection and its role in positive health; can be either positive (presence of support, acceptance, shared experience) or negative (absence)	“… knowing that there’s other people that have the same common experiences as you…is a part of wellbeing and resilience” (P3). “All in all, the school environment is a large part of my experience growing up and part of understanding who I am” (P3). “So I struggle with some things in my town… where other people say… for example, my kid is better than your kid because my kid don’t have to wear those [braces] on her feet or her legs…” (P11) “… I was excluded from other children and they have made fun of me which really affected me mentally and I didn’t have any resources to help me through it so we need resources for children to go to and show them they are not alone.” (P5)
Disability reframes positive health concepts	Refers to positive health within context of omnipresent, chronic, and lifelong impairments	“how can individuals and families affected by disability aspire to the same wellbeing as others because the perspectives on what they think what wellbeing should be are skewed by other imperatives, judgement of people who have not experienced disability, and sometimes prejudice or pity.” (P6).
Wellbeing of the family system, not just the child, affects positive health	Refers to wellbeing, including mental health concerns and/or other support needs (e.g., unmet social needs) among family system and/or person with disability; emphasis is on concept itself not as it relates to threat to capacity	“[at the start of the school year]… you don’t know… how your child’s going to be included or not included, and how is this going to impact their wellbeing and that worry and fear that the parent has… goes back to impacting the child…” (P1) “not being alone/being connected” is a key aspect of positive health, because it is wellbeing and capacity reserve and a critical part of being able to be resilient” (P6).
Resilience is a concept compelled by the world around disabled people complicating its relationship to positive health outcomes	Refers to external factors that elevate the quantity of exposures requiring adaptation to difficult or challenging life experiences	I didn’t have a choice to be a disabled mom. It was almost like if you don’t do it, your child’s going to die type of thing.” (P10). “these children [with disabilities] are resilient because… they’re fighters.” (P8) it’s all about the balance. It can be… extremely frustrating for you and your child when they’re not successful… [and you are] feeling responsible for pushing them to get as far as they can go.” (P4)
Disability lowers the baseline capacity reserve for child/adult and caregiver	Refers to external threats to capacity, such as activation energy required to perform activities of daily living, obtain services, etc. and/or internal threats (e.g., lack of mental health or other support services)—emphasis is on lack as it relates to capacity rather than the concept/need itself	“micro wounds”, “survival mode” “really hard to even think about positive health, wellbeing, resilience.” (P8) Using the analogy of “a reserve in capacities” being akin to the amount of water in a cup, a caregiver described the situation as: “My cup is half empty… I will never ever have a full cup… It goes all to my daughter. What’s left goes to my son. The tiny bit left, goes to my spouse. There’s nothing to replenish it… (P8)

Note: Participant number is indicated in parentheses following each quote.

**Table 4:** Definitions and examples of barriers to and facilitators of obtaining information about positive health outcomes for people with lived experience of disability.

Themes/Subthemes	Definition	Example
Barriers	Real or potential obstacles to obtaining information about positive health outcomes from the disability community (e.g., a lack of something)	
Ableism	Externally imposed or internally adopted negative actions, beliefs, and prejudices about disability (e.g., struggle to advocate, feel undeserving of accommodations, etc.)	“The internalized ableism that someone with a disability and even maybe the parent probably deals with is this idea of normalcy. So growing up there’s going to be barriers and the child may feel ostracized or marginalized… I don’t know, is it a negative thing to expect that state of shock when you realized I am not an able-bodied person… ”(P3)
Isolation	Lack of awareness and/or connection to others (e.g., providers, resources, community)	“I feel like I have no one to talk to about what’s going on on a daily basis with my daughter far as school and anything else.” (P11) “It was very odd and we weren’t provided a ton of resources immediately and I’m just like a crazy mom. So I did a lot of research on my own, but immediately I felt alone.” (P2)
Limited capacity	Limited capacity to participate in extra things due to services and/or social needs and psychological needs related to disability	“These parents… I’ve seen and I’ve heard they’re beaten down so much. They don’t have anything left to give…” (P8)
Unprocessed trauma	Shock, denial, grief, and/or other emotional responses associated with realizing a difference (or that you/child is different)	“I was shedding tears because… I never imagined myself, myself as a parent have to deal with a situation like this before. So every day that I look at this little girl, I am very grateful… Because she almost died for one… And I struggle with that because she wants to know why she don’t think she’s normal. So what do you tell a 9-year-old child that asks her mother, why is she not normal?” (P11)
Facilitators	An existing resource or construct that made it easier to report or evaluate positive health (e.g., the presence of something)	
Community education	Baseline education on disability for the community	“[it is] how we think about and how we talk about disability early on and that frames parents’ mindset. So, if your first interactions are negative or disability is framed in a negative way, that’s going to change perceptions very quickly.” (P1)
Financial support	Stipend or other financial compensation for participation	“So you never know what these people that you’re meeting coming into this program is dealing with because everybody’s just, yeah, financially it is a lot. It’s really, really a lot.” (P10)
Opportunity for connection	Opportunity for acceptance and connection with others (e.g., caring people, professionals, others with shared experience, support groups)	Asking the family what they may need… What is more of a strength than a weakness in your situation? … can we help you in some type of way? (P10)
Resources	Individualized supports to help with issues of capacity and resource needs—to reduce burden and increase capacity associated with participation. Resources are further defined in 1 of 3 categories.	
Practical/logistical	Resources to help navigate the healthcare system and point of contact for questions	“[Ask us:] Can we lead you to the right direction? Is this someone we can reach out to for you or give you the right resources?” (P10)
Informational/educational	Resources about (1) raising a child with disability, next steps across lifespan, how to support child coping with disability, and/or (2) how are outcomes of study being understood and applied	I remember when we got out my son’s diagnosis, no one spoke with us about it and it actually took someone outside that was his caregiver in the NICU to refer us to a place to get us the help that we needed… I can’t imagine the parent who has multiple children who’s exhausted and who has no resources… and you have to figure it out. So educating the parent [matters], even things that just say next steps, what to do, how to move.” (P2)
Psychological	Resources to address mental health needs, coping with disability	“I was shedding tears because… I never imagined myself… as a parent [having] to deal with a situation like this before… I feel like I have no one to talk to about what’s going on on a daily basis with my daughter…” (P11)

Note: Participant number is indicated in parentheses following each quote.

## Data Availability

The datasets analyzed during this study are available from the corresponding author upon reasonable request.

## Supplementary Materials

The following supporting information can be downloaded at: https://www.mdpi.com/article/10.3390/children13030430/s1, File S1: ECHO survey.

## Abbreviations

The following abbreviations are used in this manuscript:

ASD	Autism Spectrum Disorder
COREQ	Consolidated Criteria for Reporting Qualitative Research
CP	Cerebral Palsy
CPF	Cerebral Palsy Foundation
ECHO	Environmental Child Health Outcomes
IDD	Intellectual and Developmental Disability
ICF	International Classification of Function
NIH	National Institutes of Health
P	Participant number
PAR	Participatory Action Research Methodology
