# Supplementary material for: Disability Community Perspectives on Participation in Research and Studying Positive Health
Source: Children (Basel). 2026 Mar 20;13(3):430. doi: 10.3390/children13030430 (PMC13025233; doi:10.3390/children13030430)
Supplement: Supplementary file 1 [file children-13-00430-s001.zip › children-4173694-supplementary.pdf]

Appendix A

Research Inclusion & Exclusion Survey

If you are an adult or teenager or the parent or caregiver of a child with a disability, we want to know how you feel about research and how studies include people with disabilities and things that matter to them. This quiz takes less than 5 minutes to complete.

* 1. Are you:

- ◯ An individual with CP or another condition causing disability
- ◯ The parent or caregiver of a child with CP or another condition causing disability

* 2. Have you ever participated in a research study that was NOT meant to study your disability or your child's disability?

- ◯ Yes
- ◯ No

* 3. Have you or your child ever been EXCLUDED from a research study because of a disability of any kind (physical, mental, emotional...)?

- ◯ Yes
- ◯ No

The following categories are all types of environments that can have an effect on our lives and our children's. Please tell us how important you think these different things are in your life or your child's life. It could be because they are difficult to get or because they make a big difference.

* 4. Job, food, housing, and financial security

0 (least important) 100 (most important)

* 5. Education

0 (least important) 100 (most important)

* 6. Access to healthcare

0 (least important) 100 (most important)

* 7. Discrimination, inclusion – disability related

0 (least important) 100 (most important)

* 8. Discrimination, inclusion – race, ethnicity, gender related

0 (least important) 100 (most important)

* 9. Neighborhood in which you live, the physical conditions of your housing, and access to the community

0 (least important) 100 (most important)

The next questions are about the effects of the environment on our lives. The environment here means: all the world around you, close and far, physical, social, and emotional, as you saw in the questions above.

* 10. How important is it in your opinion for individuals with disabilities to be included in research that studies the effect of environment on **health**?

0 (least important) 100 (most important)

* 11. How important is it in your opinion for individuals with disabilities to be included in research that studies the effect of environment on **quality of life**?

0 (least important) 100 (most important)

* 12. How important slider is it in your opinion for individuals with disabilities to be included in research that studies the effect of environment on **resilience and happiness?**

0 (least important) 100 (most important)

* 13. The following are categories of results that many studies use to measure environment’s (job, education, access to healthcare, community) effect on people. Which ones do you think are most important to you or your child’s situation – examples are given next to each category.

- [ ] Behavioral lifestyle (sleep, diet, physical activity)
- [ ] Physical health (lung problems, asthma, heart problems)
- [ ] Developmental and mental health (thinking, seeing, hearing, moving, feeling)
- [ ] Wellbeing (curiosity, adaptability, happiness, pain management, resilience)
- [ ] Pregnancy and baby medical conditions
